# Twelve weeks of combined physical and cognitive intradialytic training preserves alertness and improves gait speed: a randomized controlled trial

**DOI:** 10.1007/s40520-023-02511-x

**Published:** 2023-07-26

**Authors:** Špela Bogataj, Maja Pajek, Katja Kurnik Mesarič, Aljaž Kren, Jernej Pajek

**Affiliations:** 1 Department of Nephrology, University Medical Centre Ljubljana, Ljubljana, Slovenia; 2 Faculty of Sport, University of Ljubljana, Ljubljana, Slovenia; 3 Faculty of Health Sciences, University of Novo Mesto, Novo Mesto, Slovenia; 4 Faculty of Medicine, University of Ljubljana, Ljubljana, Slovenia

**Keywords:** Cognitive decline, Combined intervention, Gait speed, Attention, Hemodialysis, Prevention

## Abstract

**Background:**

Hemodialysis (HD) patients often experience cognitive deficits and reduced mobility. While studies have shown promising results of physical and/or cognitive training in older adults, their effects in HD patients remain understudied.

**Aims:**

This study aimed to evaluate the impact of a 12-week intradialytic training program combining cognitive training with physical exercise on attention domains and spontaneous gait speed (SGS) in HD patients.

**Methods:**

Forty-four HD patients were randomly assigned to either intradialytic cognitive and physical exercise training (EXP group; *n* = 22) or a standard care control group (CON group; *n* = 22). The EXP group performed intradialytic cycling and tablet-based cognitive training three days per week for 12 weeks. The primary outcome of the study was performance on the computerized test battery ‘Test of Attentional Performance.’ Secondary study outcome was patient mobility assessed by the four-meter SGS. Outcomes were assessed pre- and post-intervention.

**Results:**

Significant group x time interaction was observed in alertness (F(1,41) = 6.15, *p* = 0.017) and SGS (F(1,41) = 18.33, *p* < 0.001) in favor of the EXP group. Within-group analysis revealed a significant pre–post decline in the CON group in alertness test (−26.7 s; *p* = 0.04) and an improvement of SGS in EXP group (+ 0.07 s; *p* < 0.001).

**Discussion:**

This original study demonstrated that a combined physical and cognitive intradialytic training intervention led to improvements in SGS and preservation in alertness compared to a deterioration in the CON group.

**Conclusion:**

Findings suggest that the intervention may serve as an effective tool to prevent the physical and cognitive decline in this patient population.

## Introduction

Patients with chronic kidney disease (CKD) treated with hemodialysis (HD) experience a decline in physical function and mobility, including difficulties with walking, balance, and coordination [1, 2]. This may be related to the effects of the disease itself and the treatment process, which cause muscle weakness and physical fatigue [3]. In addition, this patient population is at increased risk for cognitive impairment with significant deficits in attention and executive function [4]. Furthermore, cognitive decline in the general elderly population can affect gait parameters and a gait speed under 1 m/s associates with elevated risk for mortality and other detrimental health-related outcomes [5, 6]. When various affected cognitive components in middle-aged and older adults were analyzed, attention was the cognitive component most strongly related to gait speed [7]. Given the clear relationship between physical and cognitive performance, the impact of novel interventions targeting these two modalities should be explored to improve outcomes in this vulnerable population.

A physical exercise intervention showed to be effective in improving physical performance in HD patients [8]. Given the fact that gait speed is correlated with cognitive impairment, improving cognitive abilities could also be beneficial for improving patient’s physical performance. Nowadays, cognitive training is studied to improve physical performance-related outcomes and to counteract cognitive decline [9–11]. Most studies typically included sedentary or frail older adults as participants, and in general they have shown significant positive effects on physical performance [12–14]. The full extent of the effects of cognitive training, either alone or in combination with other non-pharmacologic approaches in HD patients remains unknown.

On the other hand, while existing literature suggests a positive effect of physical exercise on cognitive function, there is a lack of high-quality randomized controlled trial data and inconsistencies in the available evidence. Generally, the number of studies examining the effects of non-pharmacological interventions on cognitive function is low in HD population and they mostly involved interventions in the form of physical exercise. A walking program in HD patients showed an improvement in self-reported cognitive function [15] and its maintenance over a six-month period [16]. In a pilot randomized controlled trial [17], an intradialytic cycling group significantly reduced cognitive impairment compared with the control group. In contrast, intradialytic resistance exercise [18] and a chair stand exercise program [19] did not show significant effects on cognitive function. As far as the combination of cognitive and physical training is considered, there are no published studies except for an interventional pilot study in HD patients using an experimental group undergoing a cognitive training intervention, a second experimental group undergoing intradialytic cycling and a control group [20]. After three months, both intervention groups maintained the measured cognitive domains, whereas the control group showed a decline [19].

Assuming that both physical exercise and cognitive training have promising effects on cognitive performance in HD patients, it would be worthwhile to combine them. Therefore, the objective of this study was to examine the effects of cognitive training in conjunction with intradialytic cycling on cognitive and physical performance of dialysis patients. Considering that HD patients have significant attention deficits and physical performance deficits with reduced mobility, we chose attention cognitive domain and spontaneous gait speed (SGS) as key outcome variables. We hypothesized that the combination of cognitive and physical exercise interventions will reduce attention deficits and enhance gait speed, or at minimum, prevent the decline of these performance measures in a representative sample of dialysis patients.

## Methods

### Study design

The present study was a single-blind randomized controlled trial aimed to investigate the effects of a bi-modal non-pharmacological intervention on cognitive parameters and SGS in HD patients. Patients were recruited from a local dialysis center in Ljubljana, Slovenia. The inclusion criteria were as follows: HD kidney replacement therapy (duration > 3 months), stable medical condition, absence of neurological diseases, and the ability to walk independently. The exclusion criteria were: active malignant or infectious disease, uncontrolled hypertension, angina pectoris (2–4 on the Canadian Cardiovascular Society scale), heart failure (3–4 on the New York Heart Association scale), severe cognitive impairment or dementia, history of limb amputation, or any other condition that could cause the patient to be clinically unstable. The study adhered to the ethical principles set forth in the Declaration of Helsinki of 1964 and received approval from the National Medical Ethics Committee (KME0120-474/2021/4). Prior to enrollment, all participants provided written informed consent. The clinical trial was registered with ClinicalTrials.gov (NCT05150444).

### Participants

Out of the initial 72 individuals who were screened for eligibility, 44 were randomly assigned to either the intervention (EXP) or control (CON) group. Baseline characteristics of patients are presented in Table 1.

**Table 1:** Demographic and baseline clinical characteristics

	All participants (*n* = 44)	Intervention group (*n* = 22)	Control group (*n* = 22)
Age (years)	66.5 ± 11.0	65.7 ± 9.7	67.2 ± 12.5
Male sex (%)	66%	54%	77%
Height (cm)	170.3 ± 11.4	169.6 ± 12.5	171.0 ± 10.5
Weight (kg)	75.6 ± 18.3	77.1 ± 21.9	74.2 ± 14.3
Dialysis vintage (years)	6.6 ± 6.1	7.1 ± 7.6	6.1 ± 4.3
Lean tissue index (kg/m^2^)	11.9 ± 3.6	11.4 ± 2.2	12.5 ± 4.6
Fat tissue index (kg/m^2^)	13.5 ± 5.5	14.6 ± 6.6	12.4 ± 4.0
Phase angle (°)	4.4 ± 0.8	4.6 ± 0.8	4.3 ± 0.8
BIA assessed overhydration (L)	2.2 ± 1.6	1.8 ± 1.6	2.6 ± 1.6
Hemoglobin (g/L)	113.3 ± 12.4	110 ± 12.7	116.6 ± 11.4
Albumin (g/L)	36.7 ± 2.8	37.0 ± 2.2	36.3 ± 3.3
C-reactive protein (mg/L)	5.3 ± 5.5	4.4 ± 3.7	6.1 ± 6.9
Phosphorous (mmol/L)	1.6 ± 0.4	1.6 ± 0.5	1.5 ± 0.3
Systolic blood pressure (mm Hg)	156 ± 22	160 ± 22	151 ± 22
Diastolic blood pressure (mm Hg)	87 ± 11	88 ± 13	85 ± 9
Serum pre-dialysis creatinine (qmol/L)	841 ± 169	845 ± 152	837 ± 189
Urea (mmol/L)	23.1 ± 5.6	23.6 ± 4.8	25.4 ± 5.73
Davies comorbidity grade 0/1/2 (*n* (%))	0 (0)/29 (66)/15 (34)	0 (0)/14 (64)/8 (36)	0 (0)/15 (68)/7 (32)
Charlson comorbidity index	5.7 ± 2.2	5.7 ± 2.5	5.6 ± 1.8
MoCA (score)	24.7 ± 2.8	25.0 ± 2.8	24.3 ± 2.9

Values are expressed as mean ± SD, percent of subjects or index/score/grade. There were no statistically significant differences between the groups. Blood pressure was defined as the mean of the last three pre-dialysis blood pressure values. Phase angle measurements were performed with an 800μA current at a frequency of 50 kHz. *n;* number of subjects, *BIA*; bioimpedance performed using Body Composition Monitor, Fresenius AG, Bad Homburg, Germany; *MoCA*; Montreal Cognitive Assessment.

### Study flow and protocol

A detailed study protocol is described in Bogataj et al. (2022)[21]. After the screening process, all eligible patients who agreed to participate underwent baseline testing, that included tests of attentional performance and gait speed. They were then randomly assigned, using an online program (www.randomization.com), in a 1:1 ratio to either the EXP or CON group. Patients in the EXP group participated in a physical exercise and cognitive training program, while patients in the CON group received standard HD care. The intervention lasted for 12 weeks, with three training sessions per week (= 36 training sessions in total). Following the intervention, all patients were re-tested for the same outcomes as at baseline. The measurements were conducted on non-dialysis days, on the same day of the week and at the same time of day, with the same endpoint outcome assessors who were blinded to treatment allocation.

### Physical exercise training

The EXP group performed an aerobic exercise session during dialysis. This type of training was delivered in the first two hours of dialysis, three days a week for 12 weeks, on a customized bed ergometer (BedBike, Lemco, Denmark). The cycling session began with a 5-min warm-up and ended with a 5-min cool-down. In the main part, we aimed to reach 30 min of exercising with a gradual increase in duration and resistance. The resistance was implied to each individual’s capability according to the rate of perceived exertion with maintaining the intensity level of 4th–5th grades on a 10-grade Borg scale, which was shown to be effective in this patient population [8, 22].

### Cognitive training

Cognitive training was delivered on tablets through various games which target different cognitive subcategories. Each patient had their own profile on the CogniFit platform (CogniFit INC;USA), allowing the software to adjust the difficulty of the games based on individual performance to ensure that they were always being challenged without being overwhelmed. We selected the Personalized Brain Training package, which is designed to improve a wide range of cognitive skills, including memory, attention, perception, and coordination.

After the cycling session, the EXP group patients performed a 30–40-min cognitive training session. The length of the cognitive training session was based on a systematic review by Marusic et al. [11]. Research assistants supervised the sessions, helping with program initiation and ensuring the patients' understanding of the instructions for each game.

### Outcome measures

Outcomes were assessed before and after the 12-week intervention. The primary outcome of the study was assessed by the computerized test battery ‘Test of Attentional Performance’ (TAP). This was based on its reported low learning effect [23], sensitivity to physical exercise [24], and the fact that attention is one of the most impaired cognitive abilities in HD patients [4]. From the TAP test battery [25], we selected the subtests Alertness, Selective attention and Divided attention. The tests were administered to each patient individually by a psychologist in a quiet, distraction-free room. The tests were relatively quick to administer and typically took 3–5 min each.

The Alertness subtest is designed to assess an individual's level of alertness and vigilance, which are important aspects of attentional processing. The test consists of a simple reaction time task in which the subject must react as quickly as possible to a stimulus [25]. The test assesses the speed and accuracy of the participant's response.

The Selective Attention subtest assesses the ability to pay selective attention to relevant information and to suppress unwanted reactions [25]. The test requires the subject to identify a target stimulus embedded in a series of distracting stimuli.

The Divided Attention subtest assesses a person's ability to attend to and process two different sources of information simultaneously [25]. The test requires the subject to perform two tasks simultaneously, one task being visual and the other auditory. In this "dual task," the subject must discriminate a visual stimulus (recognizing a square among crosses) and an auditory stimulus (recognizing irregularities in a sequence of sounds). The test measures the speed and accuracy of the subject's responses to both stimuli and assesses his or her ability to divide attention between the two tasks.

The secondary outcome of the study was the SGS, which is currently the most used test to assess mobility [26]. Patients were instructed to walk at their normal pace over a 6-m course, with a 4-m course marked in the middle for measurement. The average time of two attempts was calculated to evaluate the patients' gait speed (m/s).

### Statistics

Statistical analysis was carried out using IBM SPSS version 29 (IBM Corporation, USA). GraphPad Prism software v9 was used for graph creation. Normality and sphericity were confirmed using Shapiro–Wilk and Mauchly's test. A repeated-measures ANOVA (2 × 2) with randomized group (EXP vs. CON) as between subject factor, and time (pre- and post-intervention) as a within subject factor was performed on TAP tests results and SGS. A paired-samples t-test was used to determine within-group differences over time. The study employed an intention-to-treat (ITT) analysis as the primary method of analysis. Cohen's d effect size (ES) was used to assess the observed differences magnitude for each group. A magnitude of 0.2 < ES ≤ 0.5 was considered small, while a magnitude of 0.5 < ES ≤ 0.8 and ES > 0.8 was treated as moderate and large [27].

The sample size calculation was performed using G*Power. It was calculated based on the results of a previous study [28] investigating the effect of aerobic exercise intervention on Alertness test results in multiple sclerosis patients. A paired t-test was conducted using the before and after results to calculate the required sample size. An alpha error probability of 0.05 and a 1-beta error probability of 0.80 were used, with an effect size of 0.565 taken from the abovementioned study. To account for an expected dropout rate of 20%, a sample size of 42 was calculated to ensure adequate statistical power to detect a difference between the two study groups.

## Results

### Tolerability, adverse events, and adherence

This bi-modal intervention in HD patients was well tolerated. No intervention-related adverse events were reported. One patient from the CON group was lost to follow-up due to transfer to another dialysis center because of colonization.

Adherence to the cycling and cognitive training programs was defined as the total number of completed sessions divided by the total number of all sessions. Adherence to cycling sessions was 79.9 ± 21.2%, with an average session duration of 37.6 ± 12.7 min. Cognitive training adherence was higher, reaching 84.2 ± 14.9% with an average session duration of 34 ± 4 min.

Reasons for skipping cycling sessions included pain, fatigue, hematoma, upper respiratory tract infection, COVID-19 infection, hypertension or hypotension, and dyspnea. Cognitive training sessions were mostly missed due to fatigue or COVID-19 isolation.

### Cognitive tests

Pre- and post-TAP scores are shown in Table 2.

**Table 2:** Pre- and post-TAP values per group

Variable	Group	Pre-test (ms)	Post-test (ms)	Pre- to post-change (95% CI)	*p* value	ES (pre–post)
Alertness						
	EXP	391 ± 117	348 ± 94	43.3 (− 8.7 to 95.3)	0.098	+ 0.37
	CON	428 ± 216	455 ± 236	− 26.7 (− 52 to − 1.4)	**0.040**	− 0.48
Selective Attention						
	EXP	472 ± 132	451 ± 94	20.4 (− 22.1 to 62.9)	0.329	+ 0.21
	CON	498 ± 206	530 ± 263	− 32.1 (− 90.9 to 26.6)	0.267	− 0.25
Divided Attention						
	EXP	597 ± 106	582 ± 112	15.2 (− 18.1 to 48.5)	0.352	+ 0.21
	CON	604 ± 132	588 ± 160	16.1 (− 36.6 to 68.7)	0.530	+ 0.12

Repeated− measures ANOVA revealed a significant main effect of group (EXP vs. CON) x time (pre- vs. post-) interaction for Alertness test results (F(1,41) = o6.15, *p* = 0.017, *ŋ*^2^ = 0.13) in favor of the EXP group. Conversely, no significant group x time interaction was observed for Selective Attention (F(1,41) = 2.31, *p* = 0.136, *ŋ*^2^ = 0.053) and Divided Attention (F(1,41) = 0.001, *p* = 0.977, *ŋ*^2^ = 0.00).

Within-group analysis showed a significant pre–post difference only for the CON group for the Alertness test score, indicating a worsening in this cognitive domain (Table 2).

Values are expressed as mean ± *sd*: Significant within-group changes are signed with bold. *ms*: milliseconds; *EXP*: intervention group; *CON*: control group; *ES*: Cohen’s d effect size; *CI*: confidence interval; *p*: level of significance.

### Spontaneous gait speed

**Fig. 1:**
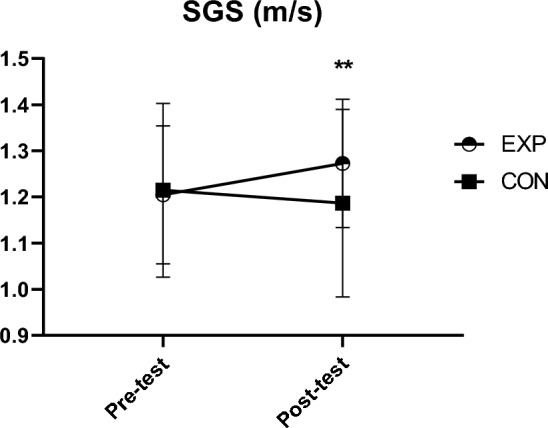
Change in spontaneous gait speed SGS, spontaneous gait speed; EXP, intervention group; CON, control group; **, significant between-group change (*p* < 0.001)

Data analysis showed a statistically significant main effect for time x group interaction (F(1,41) = 18.33, *p* < 0.001, *ŋ*^2^ = 0.31). Within-group changes are graphically presented in Fig. 1. The baseline results for the SGS were 1.21 ± 0.19 m/s for the CON group and 1.20 ± 0.15 m/s for the EXP group. After the 12-week intervention, the EXP group increased its pace to 1.27 ± 0.14 m/s (*p* < 0.001, ES = −1.16), while the CON group decreased it to 1.2 ± 0.2 m/s (*p* = 0.155, ES = 0.32).

## Discussion

This study investigated the effects of 12 weeks of combined physical and cognitive training on attention domains and SGS. Results from RM ANOVA showed a significant between-group interaction over time in alertness and SGS in favor to the EXP group. Within-group analysis in attention domains revealed a significant pre–post difference solely in the CON group in alertness test score, indicating a noticeable decline in this specific cognitive domain over the course of the study. Furthermore, the intervention resulted in a significant improvement in SGS compared with standard care.

While significant within-group improvements in attention domains were not observed, our intervention was found to be associated with the preservation of these domains. In contrast, the CON group exhibited a slight decline in selective attention and a significant decline in alertness. These results indicate that our intervention had a beneficial effect in maintaining attention abilities, providing support for its potential effectiveness in preventing cognitive decline in these domains. Our findings are consistent with gerontology studies, in which cognitive training and/or physical exercise training improved or preserved some, although not all, cognitive domains [29–31]. In a study with older adults, cognitive training showed a potential positive effect on executive function and other cognitive domains [30]. In a 6-month exercise intervention, older adults who did not exercise showed a decline in cognitive function, whereas those who participated in the exercise program maintained cognitive function over an 18-month period [31]. This study expanded previous findings on age-related cognitive decline to decline associated with end-stage renal disease, HD replacement therapy, and different age groups.

In a recent study conducted by Guo et al. (2022), attention was identified as the cognitive domain exhibiting the highest proportion of impairment among HD patients. The current study supports this finding by showing a significant reduction in alertness in the control group over a short period of time, indicating a rapid decline in this subdomain of attention. Importantly, Guo et al. (2022) also found a significant correlation between attention impairment and higher mortality rates in this population [32]. These findings highlight the potential impact of attention deficits on disease progression and prognosis, and serve as a starting point for future research investigating strategies to improve and maintain attention skills.

This is the first study examining the effects of a non-pharmacological intervention on the domains of attention in HD patients. Only one study investigated the effects of either intradialytic cycling or intradialytic tablet-based cognitive training on cognitive performance in HD patients [20]. Both intervention groups demonstrated the preservation of psychomotor speed and executive functions, in contrast to the control group, which experienced a decline. Our study aimed to exploit a synergistic effect of both interventions and to test the effects on the cognitive domain of attention using tests with high sensitivity and specificity. Our findings are consistent with the results of a study by Briken et al. [28], in which 8–10 weeks of aerobic exercise training in patients with progressive multiple sclerosis resulted in a significant improvement in alertness in the exercise group compared to the waitlist controls (*p* < 0.001).

A plethora of literature now exists linking cognitive processes, particularly executive functions and attention, with gait [33]. Improving SGS in the EXP group could potentially strengthen the evidence for a common neural substrate between these processes. The second noteworthy discovery pertains to the observed increase in gait speed by 0.07 m/s in the EXP group, which is indicative of a modest, yet meaningful and clinically significant improvement [34]. Improved SGS could have an impact on survival as gait speed has been identified as a subclinical indicator of physiological reserve and a marker of resilience to stress [35]. Previous research has shown that gait speed serves as a predictive indicator of all-cause mortality and cardiovascular events in HD patients [36].

Tests such as the Mini-Mental State Examination (MMSE) and the Montreal Cognitive Assessment (MoCA) are primarily used for screening mild cognitive impairment and dementia and may not be sufficiently sensitive to detect intervention effects [37, 38]. Additionally, these tests are prone to learning effects. Our study sought to address these limitations by utilizing specific and sensitive tests that minimize learning effects and provide more accurate assessments of cognitive function [25]. We conducted the baseline and post-test of our patients on non-dialysis days, thereby circumventing the potential confounding factors of pre- or post-dialysis fatigue effects. The patients demonstrated a high level of adherence throughout the entire study, presumably because the intervention was delivered during dialysis. Also, the intervention involved a multidisciplinary team of kinesiologists, physicians, nurses, and psychologists, which made the process more holistic and allowed for comprehensive patient care.

A limitation is that the results would benefit from replication in larger samples to improve their generalizability. Furthermore, it should be noted that the mean age of our sample was relatively low. Consequently, it is possible that older patients may exhibit lower tolerability to physical exercise interventions. Future research should include three separate groups, one participating solely in intradialytic cycling, cognitive training and the third group with both interventions. Due to the nature of the intervention, blinding of investigators and subjects was not possible. However, outcome assessors were blinded to group allocation. Consideration of these limitations helps to contextualize the results and highlight opportunities for future research to address these concerns and expand our understanding of cognitive function and mobility in this population.

Overall, incorporating both physical exercise and cognitive training into the care of HD patients may offer a comprehensive approach to improving their overall functioning. Further investigation is required to elucidate the underlying mechanisms and identify specific targets for preventive and therapeutic interventions. Additionally, there is a pressing need to develop tailored clinical care practices to help HD patients who experience cognitive and mobility impairments. Future research endeavors should also prioritize identifying risk factors associated with functional decline. By addressing these research gaps, we can advance our understanding and improve the management of cognitive and mobility impairments in this population.

## Conclusion

In summary, our original study demonstrated that a combined physical and cognitive intradialytic training intervention in comparison to the standard care led to improvements in gait speed and maintenance in alertness compared to deterioration in the control group. Additionally, the intervention used was well tolerated, confirming the feasibility and safety of the training protocol. These findings suggest that the intervention may serve as an effective tool to prevent physical and cognitive decline in this patient population.

## Data Availability

The datasets generated during and/or analyzed during the current study are available from the corresponding author on reasonable request.
